# Understanding healthcare self-referral in Nigeria from the service users’ perspective: a qualitative study of Niger state

**DOI:** 10.1186/s12913-019-4046-9

**Published:** 2019-04-02

**Authors:** Francis Koce, Gurch Randhawa, Bertha Ochieng

**Affiliations:** 1 Institute for Health Research,University of Bedfordshire, Putteridge Bury Campus, Hitchin Road, Luton, LU2 8LE UK; 2 Faculty of Health & Life Sciences, De Montfort University, Edith Murphy House, The Gateway. Leicester, Leicester, LE1 9BH UK

**Keywords:** Self-referral, Bypass, Primary healthcare facilities, Secondary healthcare facilities, Referral facilities

## Abstract

**Background:**

The by-pass of the primary level of care to the referral facilities has continued to raise concerns for the healthcare delivery system. About 60–90% of patients in Nigeria are reported to self-refer to a referral level of care. Thus, this study sought to identify the factors that influence service-users’ decision to self-refer to the secondary healthcare facilities in Nigeria by exploring the perceptions and experiences of the service-users.

**Methods:**

Twenty-four self-referred service-users were interviewed from three selected secondary healthcare facilities (general hospitals) in Niger state, Nigeria. The interviews were tape-recorded, each lasting 20 min on average. This was subsequently transcribed and framework analysis was employed for the analysis.

**Results:**

Various reasons were identified to have resulted in the bypass of the primary healthcare facilities in favour of the secondary level of care. The identified themes were organised based on the predisposing, enabling and need component of Andersen’s model. These themes included: patients understanding of the healthcare delivery system; perceptions about the healthcare providers; perceptions about healthcare equipment/ facilities; advice from relatives and friends; service-users’ expectations; access to healthcare facilities; regulations/ policies; medical symptoms; perceptions of severity of medical symptoms.

**Conclusions:**

The findings from this study call for an evaluation of the current healthcare referral system, particularly in developing settings like Nigeria and consequently the need for developing a contextual model as applicable to individual settings. Therefore, a multifaceted approach is needed to address the current concerns to ensure patients utilise the appropriate level of care. This will ensure the primary healthcare facilities are not undermined and allow the referral levels of care to live up to their mandate.

## Background

**Fig. 1:**
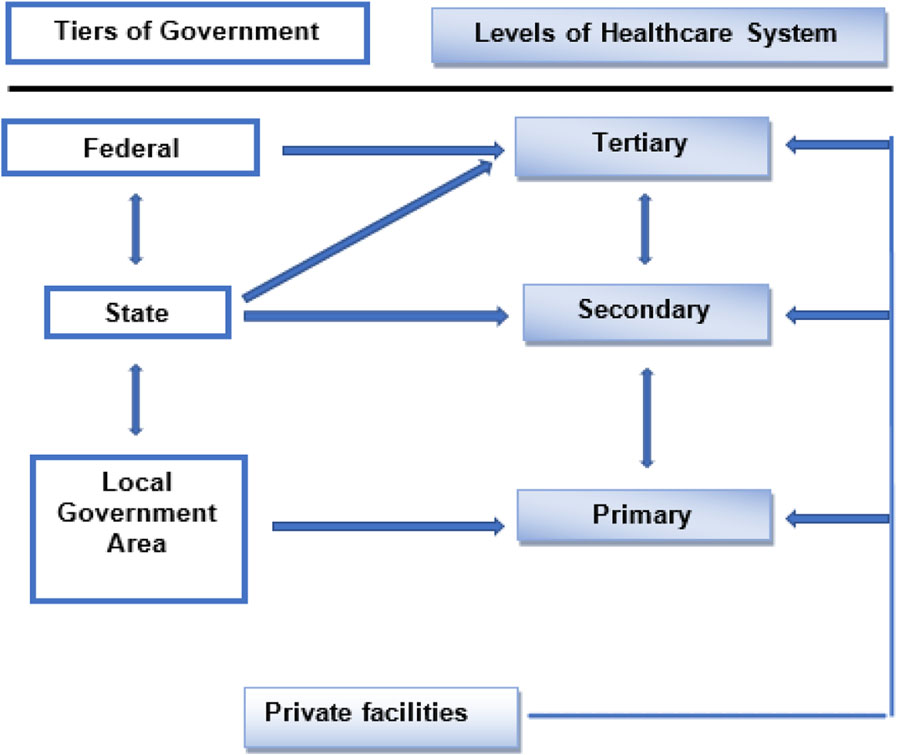
Levels of Healthcare delivery in Nigeria [67]

Following Nigeria’s independence in 1960, there have been several attempts to improve healthcare delivery [1]. Successive Nigerian government have adopted different National Development Plans (NDP) to help address development challenges in the country at different periods [2]. Some of the notable landmark in the NDP for the health sector were the 1975–80 NDP which witnessed the proliferation of healthcare facilities within communities and villages through the Basic Health Service Scheme (BHSS) [3]. The 1981–85 NDP further segmented healthcare services to be delivered across three levels of care within the public sector [3]. These are primary, secondary and tertiary healthcare system. This structure also reflects the three tiers of government in Nigeria, namely Local, State and Federal government [1, 4, 5] (see Fig. 1). Despite significant progress during these periods, there were notable deficiencies such as lack of clear policy framework, lack of manpower development and resource generation [6]. Current issues within the health sector include incessant strikes among health workers, dilapidated hospital buildings, ill-equipped laboratories, lack of healthcare financing and remuneration for the health workers [7].

In theory, the Primary Health Care (PHC) is the community entry point into the healthcare system [8]. One of the major objectives of the National Primary Health Care Development Agency (NPHCDA) is the strengthening and establishment of a standard referral system in Nigeria, through the linkage of PHC facilities with referral facilities [9]. There have, however, been challenges in accomplishing this objective. The Federal Ministry of Health, Nigeria reported a disconnection between the three tiers of healthcare delivery in Nigeria, noting that the tertiary, secondary and primary healthcare are not accountable to one another [10].

About 60 to 90% of patients reportedly bypass the PHC facilities to self-refer to the referral levels in Nigeria [11–13]. The indiscriminate use of the higher levels of care in Nigeria has led to the PHC facilities in Nigeria becoming underutilised and unrecognised, thus, wasting the resources and skills of the healthcare providers serving those facilities [1]. Additionally, referral levels have become overloaded with patients beyond the capabilities of the referral facilities. The healthcare providers in the referral facilities are also over-burdened with largely minor ailments that could have been easily addressed at PHC facilities [14, 15]. Consequently, the role of the referral facilities (secondary and tertiary facilities) in managing advanced medical conditions and engaging in research is noted to be seriously undermined in the Nigerian healthcare system [16]. However, there is a dearth of knowledge on the issue of healthcare self-referral in Nigeria, which serves to highlight the importance and timeliness of this study.

The few available literatures from the Nigerian setting such as the study carried out in a tertiary setting in Enugu state revealed that factors such as more educated persons, skilled workers, severity of ailment and presence of equipment influenced healthcare self-referral [11]. A more recent study in Oyo state, Nigeria focused on federal civil servants in a work environment. They found that the desire for quality service and competent staff were common reasons for presenting to higher levels of care [12]. Previous visit to the tertiary facility, patients seeking care for injuries and possession of health insurance were noted to influence the bypass of the primary level of care in Ghana [17]. While in Ethiopia, the lack of confidence in getting the right healthcare provider, lack of drugs and laboratory services at the primary level of care influenced patients’ decision to self-refer [18]. The above studies were however, approached in a quantitative manner. Hence, the need for this qualitative exploration to add to our understanding of the reasons underlying the motivations of service-users by-passing the primary level of care.

The current understanding regarding healthcare self-referral are largely from developed settings such as the US, the UK, Australia, the Netherlands and Japan, amongst others [19–26]. Notably, healthcare delivery differs for different settings. For example, in Nigeria, patients finance their healthcare majorly through out-of-pocket payment. In comparison with developed settings, healthcare is funded through general taxation or insurance [27]. In addition, trained medical doctors are the principal care providers within PHC facilities in most developed countries. These contrasts with the Nigerian system where most of the care providers within government owned PHC facilities are the Community Health Workers (CHWs) and nurses [28]. Given the diversity of health systems, geographical conditions and infrastructure, it is impossible to develop a global, generally applicable blueprint for referral systems [29]. Therefore, the need for a contextual approach to understand this problem is essential when we consider the Nigerian healthcare system. This study sought to understand the reasons behind service-users’ decision to self-refer by exploring the perceptions and experiences of the service-users using in-depth interviews. The research question addressed by this study is; what factors influence service-user’s decision to self-refer?

## Methods

### Theoretical model

Andersen’s model has been employed by numerous studies to understand how and why individuals utilise healthcare [30]. The original model posits that the use of healthcare service is anchored on three components namely, predisposing, enabling and need factors [31]. The predisposing component refers to variables and beliefs that exist prior to the individual’s tendency to use services [31]. Enabling components include the resources and social supports individuals have available to be able to access services. Lastly, the need component refers to the degree of illness that brings about the need to use the health service [32]. Similarly, in line with this study, findings on factors associated with healthcare self-referral from previous studies can be situated within the Andersen’s framework [21, 33, 34]. Accordingly, the purpose of the original Andersen’s model was to understand what facilitates and impedes individuals use of healthcare services, which is in tandem with the aim of this research.

### Study setting

Niger State is one of the 36 states in Nigeria. It is the largest State in terms of land mass (76,363 square km) and one of the centrally located states (North Central Region) [35]. It has an estimated population of approximately 3,950,249 [36]. Niger state is also a multicultural setting with over 15 different tribes [36]. The people of Niger State are predominantly Muslims and Christians with very few Traditional religionists and atheists. Majority of the populace in the State are farmers while others are involved in vocations such as white-collar jobs, business, craft and arts [35]. The state comprises of twenty-five Local Government Areas (LGAs). Like any other state in Nigeria, these LGAs are distributed into three senatorial districts for political reasons. The south senatorial district has eight LGAs with six general hospitals,^1^ the east senatorial district has nine LGAs with six general hospitals while the north senatorial district has eight LGAs with seven general hospitals [36, 37].

One of the healthcare objectives of the state government was to consolidate the primary, secondary and tertiary care services by way of ensuring an efficient and effective referral system [35]. Thus, between 2007 and 2015 a number of primary and secondary healthcare facilities were reported to have been constructed and renovated by the state government [38, 39].

### Recruitment and sampling

*Inclusion and exclusion criteria:* the term *self-referral* was defined as any service-user presenting directly to the General Out-Patient Department (GOPD) of a secondary healthcare facility (General Hospital) without any form of referral. Participants were 18 years and above, understood and spoke English. This study was limited to English speaking participants because Niger state has over 15 different tribes. This would have required the help of different language experts’ in conducting the interviews and translating the transcript which may have also presented the problem of losing important information due to translation. In addition, English is Nigeria’s national language and a large population of Nigerians speak and understand English. Those excluded were service-users on follow up appointment, severely ill service-users (those who could not communicate due to their ill health or were unconscious) and patients on the wards and emergency unit.

Based on the inclusion and exclusion criteria for participation, the lead researcher (FK) recruited participants from the record office of the secondary healthcare facilities, which was the first point the patients presented to at the general hospital. An information sheet was given to those who agreed to participate in the research, their contact details were also collected, and a suitable date and time was agreed upon for the interview to take place. The interviews were conducted at different agreed locations such as the participant’s residence, within the secondary healthcare facilities and a location agreed by both the participants and the researcher.

Table 1 shows lists of the different LGAs with their respective general hospitals according to their senatorial districts. One local government area with a secondary healthcare facility (general hospital) was randomly selected by way of balloting from each of the three senatorial districts. Afterwards, purposive maximum variation sampling based on age and gender was adopted to sample the participants. This was employed to generate diverse perspective regarding this topic among the service-users [40, 41]. Thus, eight participants were sampled from each of the three facilities (total of 24 participants). This entailed twelve males and twelve females. Twelve of the participants were between 18 and 40 years and the other twelve participants were 40 years and above. Other socio-demographic characteristics of the participants showed most (*n* = 20) were married. The level of education of the participants included tertiary education, secondary school qualification, primary school qualification and one of the participants reported he had no formal education. Although, the participant (SRSU16) stated he dropped out of primary school but has been involved in business which enable him travel around Nigeria. Thus, he had forced himself to learn English informally to be able to interact with people in the course of his business. Nine of the participants were civil servants (government employed), nine were unemployed, these included students, housewives or individuals in search of a job. While six of the participants were non-government employed (they were farmers, taxi driver, plumber and traders) (see Table 2).

**Table 1:** Lists of different LGA with their respective general hospitals in the three senatorial zones

LGA in South Senatorial District (Zone A) + General hospitals	LGA in East Senatorial District (Zone B) + General hospitals	LGA in North Senatorial District (Zone C) + General hospitals
Bida General Hospital Bida	Bosso	Agwara
Agaie General Hospital Agaie	Chanchaga General Hospital Minna	Borgu General Hospital New Bussa
Katcha	Gurara	Kontagora General Hospital Kotongora
Lavun General Hospital Kutigi	Paikoro General Hospital Kaffin Koro	Mariga Aminu Isa General Hospital Mariga
Edati	Rafi General Hospital Kagara	Wushishi General Hospital Wushishi
Gbako	Shiroro General Hospital Kuta	Magama (2 General Hospitals) General Hospital Nasko General Hospital Auna
Lapai (2 GH) General Hospital Lapai General Hospital Gulu	Munya	Mashegu
Mokwa General Hospital Mokwa	Suleja General Hospital Suleja	Rijau General Hospital TungaMagajiya
	Tafa General Hospital Tafa (Sabon Wuse)

**Table 2:** Socio-demographic characteristics of participants (service-users)

Identification no.	Age	Gender (M = Male; F=Female)	Occupation	Educational level	Marital status
Facility 1					
SRSU1	43	F	Government employed	Tertiary	Single
SRSU2	45	M	Government employed	Tertiary	Married
SRSU3	42	F	Government employed	Secondary	Married
SRSU4	29	M	Non-government employed	Secondary	Married
SRSU5	32	M	Non-government employed	Secondary	Married
SRSU6	33	F	Unemployed	Tertiary	Married
SRSU7	41	M	Government employed	Tertiary	Married
SRSU8	20	F	Unemployed	Secondary	Single
Facility 2					
SRSU9	29	M	Unemployed	Secondary	Single
SRSU10	41	M	Government employed	Tertiary	Married
SRSU11	42	F	Government employed	Tertiary	Married
SRSU12	41	F	Non-government employed	Tertiary	Married
SRSU13	32	F	Unemployed	Tertiary	Married
SRSU14	21	F	Unemployed	Secondary	Married
SRSU15	39	M	Government employed	Tertiary	Married
SRSU16	45	M	Non-government employed	No formal education	Married
Facility 3					
SRSU17	58	M	Non-government employed	Secondary	Married
SRSU18	30	M	Unemployed	Tertiary	Married
SRSU19	41	F	Unemployed	Secondary	Married
SRSU20	39	M	Government employed	Tertiary	Married
SRSU21	54	M	Unemployed	Secondary	Married
SRSU22	50	F	Non-government employed	Primary	Married
SRSU23	23	F	Unemployed	Tertiary	Single
SRSU24	30	F	Government employed	Tertiary	Married

Facility: Secondary healthcare facility (general hospital)

Overall 31 patients were approached to participate in this study but seven declined. Four patients that declined, specifically stated they were not interested in participating in the research when approached. Another patient stated she was too ill to participate. Though one of the patients initially accepted to participate in the study, on subsequent telephone contact to arrange a suitable date and time for the interview the patient declined to participate. Lastly, one of the patients resided in another village far away from where the hospital is located which posed difficulty in scheduling a meeting with him for the interview.

### Data collection

The data collection was carried out between 11th May 2015 and 21st June 2015. In-depth semi-structured interview was adopted to generate the data needed for this study. This approach is versatile across a range of study topics and not just important for providing information but for generating understanding as well. In-depth interviews can also explore complex and diverse patterns of behaviour, generate hypotheses and inform questionnaire development [40, 41]. Likewise, Focus Group Discussion (FGD) can generate similar data [41], however, putting this study into context, the potential difficulty anticipated from recruiting the self-referred service-users to participate in FGD was considered. This was because the service-users present to the healthcare facilities from different locations, which posed difficulty regarding the logistic of getting participants together at a particular location at the same time as required for FGD.

Lists of questions guided the interview by ensuring that the potential factors that influence healthcare self-referral to secondary healthcare facilities were captured. The semi-structured interview schedule ensured that the questions were organised but not necessarily asked in a specified order [42]. An interview guide was developed taking into cognisance the findings from the literatures and the researcher’s experiences. The interview guide was structured based on the three main components of Andersen’s model (see Table 3). There were no repeat interviews and data saturation was achieved from the 24 participants of this study who were mainly sampled based on purposive maximum variation technique for age and gender. The interviews were tape recorded and lasted on average 20 min. A 200 naira (approximately 40 pence) phone top up voucher was sent to the mobile phone of the participants on completion of the interview to appreciate them for their time.

**Table 3:** Interview guide questions

Components of Andersen’s model	Questions
Predisposing	From your understanding, can you tell me about the functions of the primary healthcare facilities (small clinics)? Can you also tell me about the functions of the secondary healthcare facilities (general hospitals)? Which level of healthcare facility (primary, secondary or tertiary) is supposed to be your first point of contact when you have any health problem? (Why do you think the facility should be your first point of contact?)
Enabling	Have you attended the PHC facility (small government clinic) in the past for any reason? -If no, is there any particular reason that made you avoid using them? -If yes, what was your experience using the facility/ services? Additional question for patients who had used the PHC facilities in the past; are there reasons that have prevented you from going back to the PHC facility for your current medical condition? How did you come to know about the secondary healthcare facility you attended? What do you think about the services provided by the secondary healthcare facility? What are the reasons responsible for you and other patients preferring to come directly to the secondary health facility (general hospital) rather than use the primary healthcare facility? Can you tell me more about other factors that you think might make patients directly present to the secondary healthcare facility (general hospital)? (Probing for the roles of opening hours, waiting time, transport, fees, healthcare providers, service provided if not mentioned) From your view as a service-user what are the likely things that will encourage you and other service-users to use the PHC facilities?
Need	What medical problem/condition brought you to the secondary healthcare facility (general hospital)? What was your thought about the problem/ what did you think was going on? (Probing for the perception of the seriousness of their condition) Did you think this problem cannot be managed at the primary healthcare facilities? Why?

### Data analysis

The tape-recorded interviews were transcribed and labelled with pseudonym. The transcripts were subsequently uploaded into the NVIVO 10 software. Framework analysis was adopted for the analysis of the data based on the 5 key stages [43], which included;

*Familiarisation with the data:* the interviews were personally conducted, tape recorded and transcribed by the lead researcher (FK). The transcripts were also read by the other members of the research team (GR and BO). All this process ensured that familiarity with the data was achieved. *Identifying a thematic framework:* themes were compared and reviewed, and consensus was reached by the team (FK, GR and BO) on merging some themes that appeared similar. *Indexing:* the themes were subsequently applied to the textual form of all the transcribed data. *Charting:* at this point a spread sheet was created, thus, the Page (P) and Line (L) numbers depicting the quotes from the transcripts of each participant that fits within the identified themes were placed in the respective cells to be able to track the account [44]. *Mapping and interpretation of data:* the themes were inspired by the original objectives of this research. Therefore, the charts were reviewed to make connections between and within participants to seek explanation for the patterns of the data.

The framework analysis employed for this study accommodates both a priori and emerging themes [43]. This makes the Andersen’s model suitable for this study by helping to provide a structure and explanation for the findings based on the components (predisposing, enabling and need factors) of the model. Despite adopting the Andersen’s components as the overarching themes for this study, the process of coding the data into categories within the NVIVO software allowed the specific themes of this study to emerge from the data. This is summarised in Table 4.

**Table 4:** Summary of generated themes and codes/nodes

Thematic categories	Organising codes
Predisposition to self-refer	
Understanding of healthcare delivery	First point of call: PHC facility Secondary health facility
	Role of PHC facility: An alternative facility to the secondary facility Closer to the people First aid and educational role Occasional and specific services
	Role of secondary health facility: Perceived as a process Referral facility Wider range of medical services
Enablers to self-refer	
Access to the healthcare facility	Distance
	Opening and closing hours
	Service fees at secondary facility
	Socio-economic status
	Waiting time
Advice from friends, relatives and others	Influenced by relatives Influenced by friends
Expectations of service-users	Negative attitude from staff at PHC
	Time wasting going to PHC facility
	Lacked trust for healthcare providers in PHC Need for supervision of the facilities
Government regulations (policies)	
Role of equipment or facilities	Absence of equipment or facilities at the PHC facilities
	Availability of basic equipment at the secondary healthcare facilities
	Lack of investigation prior treatment at PHC
Role of healthcare providers	Trained and qualified staff
	Lack of staff Level of knowledge of the staff at PHC facility
	Presence of doctors at the secondary facility
Need to self-refer	
Level of severity	Mild
	Severe
Symptoms	Tiredness, stomach ache, feverish feeling, headaches

## Results

The summary of the findings from this study are presented in Table 4.

### Predisposing factors

The predisposing component of the Andersen’s model refers to variables that exist prior to the start of the illness that describes the individual’s tendency to use services. Measures of this component are numerous, they include age, sex, race, religion and beliefs pertaining to health and illness [45]. Due to the qualitative nature of this study whereby purposive sampling was employed and the use of a small sample size, the relevance of the effect of socio-demographic variables cannot be ascertained as these were not factors participants directly talked about. Hence, age, education, marital status, employment status and gender were captured as socio-demographic characteristics but not as a theme. Only one theme emerged from the analysis regarding predisposing factors, this relates to the participants understanding of the healthcare delivery system.

#### Understanding of healthcare delivery

The responses and meanings the participants talked about, specifically regarding what they felt were the roles of the primary and secondary healthcare facilities when they were asked the question are presented here with supporting quotes. Varying accounts were presented by the participants regarding their understanding of the healthcare delivery system which may have prompted bypassing the PHC facility to a higher level of care.

Some participants identified the PHC facilities as primarily for first aid measures:

*“It’s just like a temporary first aid measure, from the way I see it, due to the level of facilities and other factors that are involved. So, for me, I just feel it’s set up for basic everyday minor ailments but nothing serious”. SRSU11, P1, L11-16*

Some noted the PHC facilities were meant to be proximal to people, while others believed that the PHC facilities were for specific groups of people or for the rural population.

*“They are the closest stage that people can run to at any time, because they are located close to them”. SRSU13, P1, L13-15*

“*I can say they are provision of medical services channelled to rural people”.* *SRSU15, P1, L10-12*

The participants, however attached broader roles to the secondary healthcare facilities which might have prompted their use, noting that in addition to pregnant women being catered for at the secondary healthcare facilities, investigations such as blood tests, scanning and surgeries can be conducted within the secondary healthcare facilities.

*“There are more equipment at the general hospital compared with the clinic (PHC facility), because at the clinic (PHC facility) there are no labs. While here (secondary health facility), they will take your blood for test and check various problems”.* *SRSU18, P2, L49-54*

The participants also recognised that prior to attending the secondary healthcare facility, one needs to be referred from the primary level of care.

*“The function of the general hospital is that, if, maybe you present to the primary healthcare and you are treated but still do not feel well then you can be referred to the general hospital”. SRSU10, P1, L25-29*

However, some participants held the notion that their first point of contact with the health system should be the secondary level of care.

*“For me, I think the secondary (…) because there are more hands there. At the primary you basically see maybe one or two people. The place is always empty”.* *SRSU11, P1, L43; P2, L49-56*

### Enabling factors

Andersen and Newman described the enabling components as a condition which permits an individual to act on a value or satisfy a need regarding health service use [47]. The enabling conditions are measured by taking into cognisance an individual’s income, health insurance coverage, rural-urban community, available healthcare providers and healthcare facilities and how they are structured to provide services. Other factors also recognised are extent and quality of social relationships, health policies and waiting time [31, 32]. Therefore, the individual’s personal experiences with the healthcare system that reflected the above mentioned features were captured within this component.

#### Role of healthcare providers

In addition to the shortage of healthcare providers reported in the PHC facilities, participants also held the notion that the staff in the PHC facilities lacked the required medical knowledge to care for them. Some participants pointed out that the cadre of healthcare providers in the PHC facilities are principally CHWs and nurses. Thus, voicing their lack of confidence with the expertise of this group of healthcare providers.

*“In primary healthcare facilities, you meet nursing officers and may be some community health workers attending to patients. I can say they have little knowledge about certain medicines. That’s the reason I prefer going to the general hospital”.* *SRSU15, P2, L56-63*

The preference by participants to be seen by a doctor and the perception that they were more likely to see a doctor at the secondary healthcare facilities prompted the bypass of the PHC facilities for some participants.

*“As I have rightly said, another factor is the issue of qualified doctors, who should be employed in those primary healthcare (…). Had it been we have a specialist doctor here (PHC facility), there will be no need for me to go to that general hospital.” SRSU5, P7, L314-316, L321-324*

#### Role of equipment or facilities

Emphasis on facilities was not only narrowed to equipment required for clinical investigations but other components, such as the structure of the building and aeration of the facilities were also mentioned. Thus, the PHC facilities were seen as lacking in these aspects while the general hospitals were perceived to have these basic amenities and consequently likely resulted in the bypass of the PHC facilities.

*“…there (PHC facilities), they don’t have equipment, that’s just the fact.”* *SRSU6, P4, L162-169*

*“Actually, the services there (secondary health facility) is perfect. Why? Because they have good doctors and the hospital itself is well organised. You know when we get to hospital as a patient and we discover that there is light, fan and the breeze is blowing, it eases the pains and sickness from the body. But when you go to a facility where there is no light, the heat will not allow you to be comfortable, you know. So, on getting to that place (secondary health facility), the environment itself makes me feel better”. P4-5, L189-201.*

Remarkably, participants spoke with keen interest to identify what is wrong with them based on objective findings from investigations conducted rather than being placed on medications or treatment based on a subjective diagnosis, which they associated with the PHC facility.

*“You know as I said earlier on, at the primary healthcare, tests are not ordered for you. You are just placed on drugs without knowing the problem you are having. They treat you through signs and symptoms only. So, that’s the reason I have to boycott the primary healthcare and go directly to the general hospital”.* *SRSU15, P5, L225-232*

There appears to be a connection between the inability of the PHC facilities to conduct a test prior to administering medications and the participant’s notion of lack of equipment in those facilities.

#### Advice from friends, relatives and others

Though most (*n* = 20) of the participants interviewed in this study were married, however, irrespective of this and the other socio-demographics factors such as age, educational level, occupation and gender, participants spoke about receiving advice from friends or relatives as one of the reasons to bypass the PHC facilities. For example, one of the participants (SRSU16) noted that he was directed to present at the general hospital by his brother.

*“One of my brothers told me to come to the general hospital in ... that I should come and just see what they will do for me”. SRSU16, P2, L89-92*

Another participant noted that he takes it upon himself to direct patients he knows to present at the secondary healthcare facility and sometimes makes himself available to take them there.

*“I prefer going to that Kaduna Road, since I found it (secondary healthcare facility), I like going there. So, anybody that is sick, any of my friends, I say let’s go. I can even volunteer and drive the person to the place (secondary healthcare facility)”.* *SRSU4, P5, L227-232*

#### Expectations of service-users

Participants also spoke about issues bordering on their expectations from the healthcare facilities that influenced their decisions to seek care at the secondary level of care.

Some participants held the view that they were unlikely to get what they wanted at the PHC facilities and thus, termed it a waste of time going to the PHC facilities.

*“You just go there (PHC facility) and waste your time, so I think I prefer to go to where I am sure I am getting what I want because it’s just like when you go to a store to buy something, you go there the first day they don’t have what you want, the second time, the next time you won’t bother because it’s like waste of time”.* *SRSU11, P2-3, L85-93*

For one of the participants, the perception of the use of the PHC facilities as a waste of time was also tied to the fact that the healthcare providers designated to a particular PHC facility may not be found at the facility. The participant further stated that the healthcare providers may be engaged with their own personal activities, such as farming, thus, leaving the service-users with no choice than to seek care elsewhere.

*“You went there (PHC facility) and you were told he went to the farm, (…). Will you waste your time and wait for that person (healthcare provider) again? Maybe before he comes, he is already tired, he cannot even listen to you very well and accommodate you”. SRSU21, P3, L123-124, L126-130*

Nevertheless, the supervision of the PHC facilities was highlighted as a way of monitoring the activities rendered at the PHC facilities, which was thought could also impact on the way the healthcare providers discharge their duties.

*“You know, there is problem, a big problem. The government should at least provide a monitoring team (…). Yes, to monitor all this primary healthcare”.* *SRSU5, P10, L453-455, L457-458*

#### Government regulations (policies)

Participants’ opinion was also sought regarding the institution of stringent government regulations to control patients’ use of the different levels of healthcare facilities. Most participants however noted that the enactment of any policy with the current state of the PHC facilities in Nigeria will not be a good idea. One of the participants (SRSU13LAP) stated that she would rather use the available private facilities than use any of the government healthcare facilities.

*“If it is possible that all the care that you can get in the general hospital is available in primary healthcare, I think its ok by me. But if there is a policy, without repairing the primary healthcare first, I think I will rather use the private health facilities. I will not even go to both the general and primary healthcare, I will just go to private hospital”.* *SRSU13, P8, L362-370*

#### Access to the healthcare facility

Participants also discussed issues around access to healthcare facilities which impacts on their decisions to bypass their primary level of care. They highlighted reasons such as the distance to the healthcare facility, fees charged at the facilities, waiting time, opening and closing hours and socio-economic factors.

##### Socio-economic status

The use of the different levels of healthcare facilities was equated with an individual’s socio-economic status. The PHC facilities were viewed as lesser facilities and thus, only deemed appropriate for the poor, which might have also influenced patients’ use of the facilities.

*“Someone might be a civil servant who is highly paid. He works in Abuja or maybe he is a senior civil servant. He will feel that his status has gone above going to a primary healthcare level”. SRSU7, P6-7, L294-298*

##### Service fees

Most participants across the different socio-demographics addressed service charges at the different level of healthcare facilities. For most participants, the goal was to find a solution to their medical problems and were not necessarily concerned about the fees they must pay at the healthcare facility.

*“Since I will get the best result I need at the general hospital, I don’t mind the cost. Despite the expensive nature of the general hospital… I will still go for the general hospital”. SRSU15, P6, L274-277*

##### Distance

The role of distance to the healthcare facility was highlighted by some of the participants. For some, the proximity of the secondary healthcare facility to where they live was a source of motivation to seek care at the secondary level.

*“Because the general hospital is in town and the PHC facility is far away. In fact, from here, you can trek to the market. Even from the PHC facility you can also trek too, but the general hospital is a bit closer to my house than that one (PHC facility)”.* *SRSU12, P4-5, L195-201*

For other participants, distance was not a factor but rather the need to find a solution for their medical concerns.

*“Well, certainly, the issue of distance to me, to a patient does not matter, because when an individual is sick, he/she is looking for a place where he/she will be cured”.* *SRSU2, P6, L266-269*

##### Opening and closing hours

Participants also mentioned the opening and closing hours of the different levels of facilities to likely impact on their use. Participants were concerned about the unpredictable nature of the operational hours of the PHC facilities. They also made their fears known regarding the possibility of fatal health consequences in the event they present to the PHC facility and found no one to offer them medical help.

*“The place (PHC facility) is always empty and the timing too. Most of the time you go there very early or you go for an emergency, they don’t come till after a while. So, are you going to wait there? If you are dying, you would have been dead before they come”. SRSU11, P2, L51-56*

Likewise, participants were also aware that the secondary healthcare facilities provide 24-h service, which appeared to impact on their choice for going directly to the secondary healthcare facility.

*“At the general hospital, healthcare providers are there 24-hours. One goes, and another person takes over. There is no time you come to the general hospital and you will not see someone to attend to you, even when the doctor is not around”.* *SRSU22, P6, L256-260*

##### Waiting time

The waiting time to see a healthcare provider was also highlighted among participants. The participants mentioned that the waiting time to see a healthcare provider at the secondary healthcare facility was longer compared to the PHC facility. They however, added that they were more comfortable waiting for a longer duration to be attended to at the secondary healthcare facility, than going to the PHC facility which is likely linked to receiving care from doctors at the secondary level.

“I don’t mind even if I am going to wait the whole day. Provided I get the best for my health, I will wait”. SRSU1, P8, L431-443

### Need factors

Andersen noted that the need factors can be viewed as perceived or evaluated need. He pointed out that the perceived need involves how individuals view their general health and functional state. He also added that symptoms the individual experiences and whether or not they judge their problem of sufficient importance and magnitude to seek professional help may be regarded as perceived need [31, 47]. Whereas, the evaluated need is a healthcare providers’ judgement or diagnosis of an individual’s medical condition necessitating their need for care [47]. However, only the perceived need was explored in this study due to the ethical and practical difficulty of assessing the evaluated need.

#### Medical symptoms

Participants spoke about the symptoms that necessitated them to use the secondary healthcare facilities. Their symptoms varied, this included feelings of tiredness, stomach ache, feverish feeling, headaches, breathlessness, dizziness and ‘heart burn’ among others.

*“The problem that brought me here is that I am feeling pain at the side of my stomach”. SRSU16, L109-111*

*“My problem is one thing, always if I am sitting and not working, I will be sleeping”.* *SRSU18, L292-294*

*“I do have heartburn and it turns my intestine”.* *SRSU24, L99-101*

Different symptoms necessitated the participants to bypass the PHC facilities to the secondary level of care, despite the likelihood that some of the symptoms might have been well managed at the PHC facilities, which reflects the indiscriminate use of the referral facilities.

#### Severity of symptoms

Participants’ perceptions of the seriousness of their health conditions were also noted. Some perceived their medical conditions as mild, while for others they felt it was severe enough to warrant presentation at the secondary healthcare facility.

##### Mild

For example, one of the participants (SRSU12), only needed re-assurance about her condition but still decided to present at the secondary level of care.

*“No, it is not that the condition is serious. I just want to, I want to know the month that I took in, to know when I am expecting my baby, just to be sure.”* *SRSU12, P3, L140-143* 167

*“I think I was a bit down, so it wasn’t like any serious major ailment”.* *SRSU11, P3, L116-117*

##### Severe

Other participants perceived their medical conditions as severe. This was attributed to varying reasons as experienced by the participants. For example, one of the participants claimed to have lost some weight which he termed to be a serious symptom for him.

*“It’s a serious condition because the way I am seeing my health, I am not like before... I used to be someone very huge but now I am losing weight”.* *SRSU17, P5, L229-233*

## Discussion

### Predisposing factors

The participants had different levels of understanding regarding the roles of the different levels of healthcare facilities, which may have impacted on their decisions to seek healthcare at the secondary levels (general hospital). Some participants perceived the PHC facilities as facilities for rural settings which they associated with the poor. Findings from this study mirrored that of related studies. For example, some of the barriers to utilisation of the PHC facilities identified by a study in the UK, included the lack of awareness by service-users of the services that GP practices offered [48]. It was also noted that the service-users did not actually understand how the healthcare system operates and did not know about alternative services [48]. Nevertheless, contrary to the findings above, in France, it was ascertained that the patients interviewed chose the emergency department of a referral facility as discerning health consumers, because the patients were well informed about the healthcare system and the primary care services available to them [49]. Therefore, they were able to identify possible alternatives, and consequently translated their assessments into a choice to use the referral facility. Nevertheless, one of the suggestions proposed by the healthcare providers, was the need for patient’s education regarding appropriate use of the healthcare services to assist them make more rational decisions [49].

### Enabling factors

Findings from this study revealed that healthcare providers play a pivotal role in the decision making of the service-users to utilise the referral facilities. Generally, the participants perceived that the PHC facilities had shortages of healthcare providers. They also remarked that occasionally the healthcare providers in the PHC facilities do close their facilities to attend to their own personal needs, such as going to their farms rather than attending to patients. Evidently, the problem of lack of staff in the PHC facilities is not peculiar to only the Nigerian healthcare system; a similar finding was reported in Tanzania in a community-based study of four Focus Group Discussion (FGD) to explore caretakers’ perceptions and expectations of services offered at PHC facilities [50]. They found that a common perception was the claim that there was insufficient staff at most facilities to provide the expected services. Additionally, this was furthermore aggravated by the frequent absenteeism of the staff [50]. The absence of attending doctors in the PHC facilities was also a common theme in the findings obtained from a semi-structured interview conducted among service-users in Saint Vincent and the Grenadines (SVG) [51].

Likewise, the perceptions of the participants regarding the different cadre (doctors, community health workers and nurses) of healthcare providers revealed they preferred to be seen by doctors who were readily available at the referral facilities. Thus, prompting the decision to bypass the PHC facilities. Similarly, in the UK, some service-users felt that they would be treated by practitioners more qualified than their general practitioner at the emergency department, which prompted them to bypass their local healthcare practice [52].

It was apparent from the qualitative findings of this study that participants’ perceptions regarding equipment/facilities was not only tied to the equipment required to make a diagnosis or conduct a test, but also the presence of amenities, such as light, water and the general environment of the facilities. Participants also placed emphasis on the need to have investigations performed to ascertain their specific medical problem before they were offered medications. However, it was highlighted that investigations are scarcely conducted at the PHC facilities. This was also a major finding among the caretakers of children under-five in Tanzania who wanted to know what was wrong with their children before they were given treatment. They were disappointed because the common practice was that the healthcare providers instituted treatment without investigation [50]. This was contrary to the referral facilities where tests were perceived to be carried out immediately [53]. Nevertheless, participants’ perceptions of their need for certain investigations is not always accurate, as not every condition warrants a test [54].

Also revealed from this study was that participants sometimes tend to consult and listen to their relatives or friends when faced with health needs. Similarly, it was also discovered that the idea to circumvent the PHC facilities to a higher level of care was a decision shared and encouraged by others, such as the participants’ families and friends [51]. This was also in tandem with the finding reported in the US [55].

Based on the perception that the participants were unlikely to get what they wanted at the PHC facilities, it was perceived as a waste of time to present at the PHC facilities, these in turn leads to loss of confidence in the PHC facilities [56]. This was also a similar finding for other studies, whereby the patients expected that their GPs would send them to the referral facilities and thus, personally decided to take that initiative [22, 52, 57].

Access to healthcare facilities was a common theme identified in the qualitative aspect of this study as a possible reason for bypassing the primary level of care. This theme was observed to have multi-faceted dimensions. For example, the participants highlighted the socio-economic status of patients as a potential factor for utilising either the primary or secondary healthcare facility. They perceived that the wealthier patients were more likely to attend the higher level of care. This assumption may be common to the Nigerian healthcare system and other similar healthcare systems, where healthcare services are predominantly paid for by out-of-pocket. Most participants felt the cost of healthcare was higher at the secondary healthcare facilities when compared to the PHC facilities. Despite this assumption, the participants still felt the need to use the referral facilities. In Namibia, it was learnt that the perception that the cost of care was relatively low at the referral facility prompted their use [58]. Similarly, in Australia, the lack of charges to see a doctor at the emergency department prompted patients to self-refer [59]. Notably, funding for healthcare systems differs for different settings, which could impact on how patients use the healthcare services available to them.

The inconsistencies of how the PHC facilities operates, coupled with the understanding that the secondary level of care is in operation 24-h a day appeared to favour the use of the secondary level of care. Nevertheless, there was general perception that the PHC facilities lacked proper supervision which has degenerated into the irregular operation of the facilities. Similarly, it has been highlighted by other studies that service-user’s inability to use their PHC facilities during regular opening hours was due to conflicts with their work schedule which prompted them to present to the referral facility [60–62].

The operation of healthcare systems in most settings is primarily regulated by the government of that country; consequently, the government have a crucial role to play in ensuring effective healthcare delivery. Accordingly, the participants in this study recognised the need for the government to be involved if an effective referral system is to be achieved in Nigeria. However, participants noted that for any government policies to be adhered to with regards to self-referral, the PHC facilities needs to operate at their expected standard. For others, an unfavourable policy meant a total boycott of the government owned healthcare facilities in favour of the private healthcare facilities. In France, it was reported that healthcare providers suggested the need to impose financial penalty on patients who inappropriately use referral facilities [49]. In the Netherlands, it was found that only about 30% of the self-referred patients were unwilling to pay the suggested amount to self-refer to the emergency department [21]. Thus, adequate evaluation and care is needed in instituting financial penalties in different context.

### Need factors

Different medical complaints necessitated participants’ bypassing the PHC facilities. Some of the symptoms included feelings of tiredness, stomach ache, feverish feelings, headaches, breathlessness, dizziness and ‘heart burn’. Likewise, other related studies noted that their participants attended referral facilities with different medical conditions [51, 60, 61, 63]. Symptoms experienced by the participants were perceived as severe for some participants and mild for others. Findings from related studies also revealed that patients bypassed the primary level of care irrespective of the perception of the severity of their symptoms [46, 54, 55].

Given the limitation of a qualitative study such as the sample size and sampling technique for this study, the findings from this study cannot be generalised beyond the examined population. Therefore, extending this research to include non-English speaking population and different region in Nigeria may help identify any geographical differences and consequently, help the government tailor their policies accordingly, if indicated. Another limitation of this study was its concentration on only the service-users from the secondary level of care and not including the tertiary level. In addition, current quantitative studies have mainly concentrated on looking at how specific factors impact on healthcare self-referral. Therefore, future quantitative studies may also consider looking at how different factors interact/ relate with one another to impact on healthcare self-referral to further advance our knowledge on this topic.

## Conclusion

Factors related to healthcare self-referral takes different forms based on the context. These contexts are in direct relation to the variations observed in the operation of the healthcare systems in different countries [64]. Accordingly, the findings from this study ignites the debate on the need for evaluation of the current model of the healthcare referral system especially for developing settings like Nigeria. Notably, the PHC concept has evolved over many decades and differs between industrialised and developing countries [65]. Major interest following the Alma-Ata Declaration of 1978, has been to take note of conflicting concepts, policies and processes in the implementation of the PHC concept in various parts of the world [64]. What has been considered as PHC in well-resourced contexts has been oversimplified in settings where resources are constrained. For example, PHC in well-resourced settings is associated with physicians who specialise in family medicine or General Practice (GP), while in developing countries it is synonymous with low technology, non-professional care [66]. In addition, healthcare delivery is mainly out-of-pocket payment for developing settings as compared to well-resourced setting with different forms of insurance and payment methods [27].

Therefore, some of the reasons for bypassing the primary level of care were contextual to the setting of this research which reflects the organisation and operation of the healthcare system in this setting. These factors, however, interact and impact on patient’s decision to bypass the primary level of care. Based on the findings, there is need for a multifaceted approach to ensure patients utilise the appropriate level of care to avoid undermining the primary healthcare facilities and allowing the referral levels of care to live up to their mandate. This should include maintaining the primary healthcare facilities at an operational level by equipping them with the necessary facilities. The need for a contextual model of financing the healthcare system is also essential rather than out-of-pocket payment. Augmenting the expertise of the PHC facilities with doctors should be considered. Educating the patients on the appropriate facilities to utilise is also important and overall the efforts of the government should be tangible by enacting appropriate policies.

## Abbreviations

CHW: Community Health Worker
FGD: Focus Group Discussion
LGA: Local Government Area
PHC: Primary Health Care
SRSU: Self-Referred Service User
UK: United Kingdom
US: United States
WHO: World Health Organization

## Footnotes

1 General hospitals are secondary healthcare facilities in Nigeria.
